# Phenotypic zinc resistance does not correlate with antimicrobial multi-resistance in fecal *E. coli* isolates of piglets

**DOI:** 10.1186/s13099-019-0342-5

**Published:** 2020-01-21

**Authors:** Fereshteh Ghazisaeedi, L. Ciesinski, C. Bednorz, V. Johanns, L. Pieper, K. Tedin, L. H. Wieler, Sebastian Günther

**Affiliations:** 1 Institute of Microbiology and Epizootics, Freie Universität Berlin, Robert-von-Ostertag-Str. 7-13, 14163 Berlin, Germany; 2 Institute of Chemical Physiology, Ludwig-Maximilians-Universität, Veterinärstr. 13, 80539 Munich, Germany; 3 Robert Koch Institute, Nordufer 20, 13353 Berlin, Germany; 4 Institute of Veterinary Epidemiology and Biostatistics, Freie Universität Berlin, Königsweg 67, 14163 Berlin, Germany; 5 Institute of Pharmacy, University of Greifswald, Friedrich-Ludwig-Jahn-Straße 17, 17489 Greifswald, Germany

**Keywords:** Antimicrobial resistance, Zinc, Co-selection, *E. coli*, Feed supplementation, Pigs

## Abstract

**Background:**

Following the ban on antimicrobial usage for growth promotion in animal husbandry in the EU, non-antimicrobial agents including heavy metal ions (e.g. zinc and copper), prebiotics or probiotics have been suggested as alternatives. Zinc has extensively been used in pig farming, particularly during weaning of piglets to improve animal health and growth rates. Recent studies, however, have suggested that high dietary zinc feeding during weaning of piglets increases the proportion of multi-drug resistant *E. coli* in the gut, contraindicating the appropriateness of zinc as an alternative. The underlying mechanisms of zinc effects on resistant bacteria remains unclear, but co-selection processes could be involved. In this study, we determined whether *E. coli* isolates from intestinal contents of piglets that had been supplemented with high concentrations of zinc acquired a higher tolerance towards zinc, and whether multi-drug resistant isolates tolerated higher zinc concentrations. In addition, we compared phenotypic zinc and copper resistance of *E. coli* isolates for possible correlation between phenotypic resistance/tolerance to different bivalent ionic metals.

**Results:**

We screened phenotypic zinc/copper tolerance of 210 isolates (including antimicrobial resistant, multi-drug resistant, and non-resistant *E. coli*) selected from two, independent zinc-feeding animal trials by determining a zinc/copper minimal inhibitory concentration (Merlin, Bornheim-Hersel, Germany). In both trials, groups of piglets were supplemented either with high dietary zinc (> 2000 ppm) or control (50–70 ppm, background) concentrations. Our observations showed that high concentration zinc exposure did not have an effect on either zinc or copper phenotypic tolerance of *E. coli* isolates from the animals. No significant association was found between antimicrobial resistance and phenotypic zinc/copper tolerance of the same isolates.

**Conclusion:**

Our findings argue against a co-selection mechanism of antimicrobial drug-resistance and zinc tolerance after dietary zinc supplementation in weaning piglets. An explanation for an increase in multi-drug resistant isolates from piglets with high zinc dietary feeding could be that resistant bacteria to antimicrobial agents are more persistent to stresses such as zinc or copper exposure.

## Background

The administration of antimicrobial growth promoters in animal husbandry has been prohibited in the EU since 2006 [1]. As alternatives to the application of antimicrobials, non-antimicrobial substances including heavy metal ions like zinc and copper, prebiotics or probiotics have been suggested to improve animal health and growth rates [2–5]. Zinc is one of the compounds widely used in the pig farming industry to overcome problems during weaning of piglets, including infections caused by pathogenic *E. coli* [6–9]. The essential trace elements zinc and copper are both involved in numerous physiological and cellular functions in all organisms [10–12]. Zinc concentrations and resistance are highly regulated through uptake and efflux mechanisms in different organisms [11, 13]. However, recent studies have suggested that feeding zinc in high concentrations during weaning of piglets increases the proportion of multi-drug resistant *E. coli* in the gut of the piglets [14–19]. The enhancement in the spread of antimicrobial resistance by the use of zinc confounds the usefulness of zinc supplementation in piglets and raises the question as to the underlying mechanisms of this observation.

One possible mechanism could be co-selection for both heavy metal/biocide and antimicrobial resistance, either in the form of co- or cross-resistance [16, 20–22]. Cross-resistance occurs as a result of physiological adaptations and affects susceptibility to different compounds, for example through efflux pump regulation or changes in cell wall permeability [23]. Co-resistance phenomena include changes involving genetic linkage of different genes encoding resistance to different classes of antimicrobials [20, 24]. A number of different studies have described possible mechanisms for co-selection of antimicrobial and heavy metal (zinc) resistance [16, 25–29]. Physiological coupling, genetic coupling and linked/co-localized resistance genes on mobile genetic elements have been suggested as possible mechanisms of both cross- and co-resistance [19, 23, 28, 30–32]. Zinc dependent beta-lactamases, effects of zinc on ampicillin stability or bacterial conjugation rates, and class 1 integrons (involved in co-selection) proximity to genes coding the efflux pump CzcA have been proposed as mechanisms involved in simultaneous reduction of susceptibility to antimicrobials and zinc/copper [19, 33–37]. Both intrinsic and acquired resistance mechanisms including efflux pumps and cellular detoxification of high concentrations of copper in bacteria have been reported in different studies [10, 38–40]. In addition to zinc, copper has also been suggested to contribute to antibiotic resistance in gram-negative and positive bacteria [28, 41, 42].

In this study, we tested the hypothesis that the increased antimicrobial resistance of *E. coli* isolates observed in weaning piglets fed with high zinc concentrations is caused by co-selection via phenotypic zinc tolerance. For this purpose, we used selected isolates [including antimicrobial resistant, multi-drug resistant (MDR), and non-resistant/susceptible (S) *E. coli*] and screened the level of their phenotypic zinc tolerance by determining a zinc minimal inhibitory concentration. Isolates originated from two, independent zinc-feeding trials of piglets with two different sampling schemes performed by our group over a period of 5 years. In both trials, groups of piglets were administered either high concentrations of zinc (> 2000 ppm) or a background control (50–70 ppm). From both feeding groups, we determined whether feeding of zinc resulted in higher proportions of phenotypically zinc resistant *E. coli*, and whether multi-drug resistant isolates also tolerated higher zinc concentrations, indicative of a co-selection process. In addition, we also compared phenotypic zinc resistance of these isolates with their phenotypic copper resistance values to determine whether there is a correlation between phenotypic resistance/tolerance to different bivalent ionic metals.

## Results

### 1. Phenotypic antimicrobial resistance

**Fig. 1:**
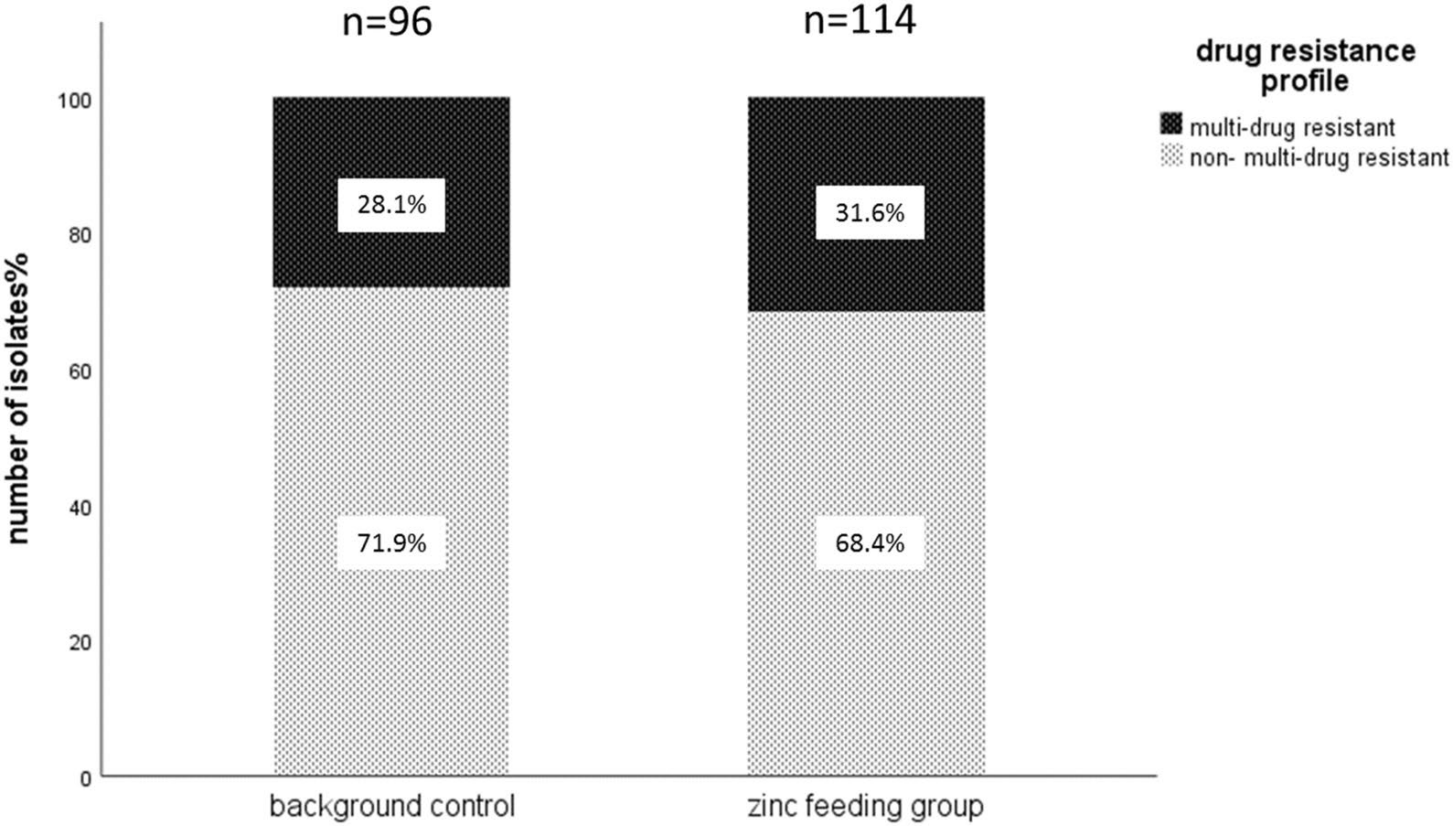
Distribution of selected multi-drug resistant (MDR) and non-multi-drug resistant (NMDR) isolates in zinc and control groups. Out of a total of 210 isolates from both zinc trials, 36/114 (31.6%) multi-drug resistant (MDR) isolates were isolated in the zinc supplemented group (54.3% of total isolates), and 27/96 (28.1%) were found in the control group (45.7% of total isolates)

Out of 210 preselected isolates collected during two, independent zinc feeding trials of piglets, 114 isolates belonged to zinc feeding groups (54.3%) and 96 isolates were from control feeding groups (45.7%). From the total number of tested *E. coli*, 63 isolates (30%) were found to be multi-drug resistant (MDR). The resistance pattern of MDR isolates always was a combination of beta lactamases (ampicillin or cefotaxime), tetracyclines (tetracycline), aminoglycosides (streptomycin) and sulfonamides (sulfamethoxazole/trimethoprim). There was no significant difference in the number of MDR isolates between the selected isolates from zinc and control groups of the feeding trials using chi-square test (Fig. 1; P-value = 0.586). Likewise, there was no significant difference in the number of resistant isolates (R) und susceptible (S) in zinc and control groups (P-value = 0.299). The number of resistant isolates to at least one antimicrobial agent was 124 (59%) of all 210 tested isolates.

### 2. Zinc tolerance (MIC)

All 210 *E. coli* isolates examined in our study were tolerant to 64 µg/ml zinc chloride (break point 128 µg/ml–1 mM) (lower cut-off). The highest tolerated zinc chloride concentration was 256 µg/ml (break point 512 µg/ml–3.7 mM). This includes only 33.3% of isolates (n = 70) (upper cutoff). The largest proportion of isolates (64.3%) showed a medium level of tolerance to zinc chloride at 128 µg/ml (break point 256 µg/ml–1.9 mM) which comprises 135 isolates.

**Fig. 2:**
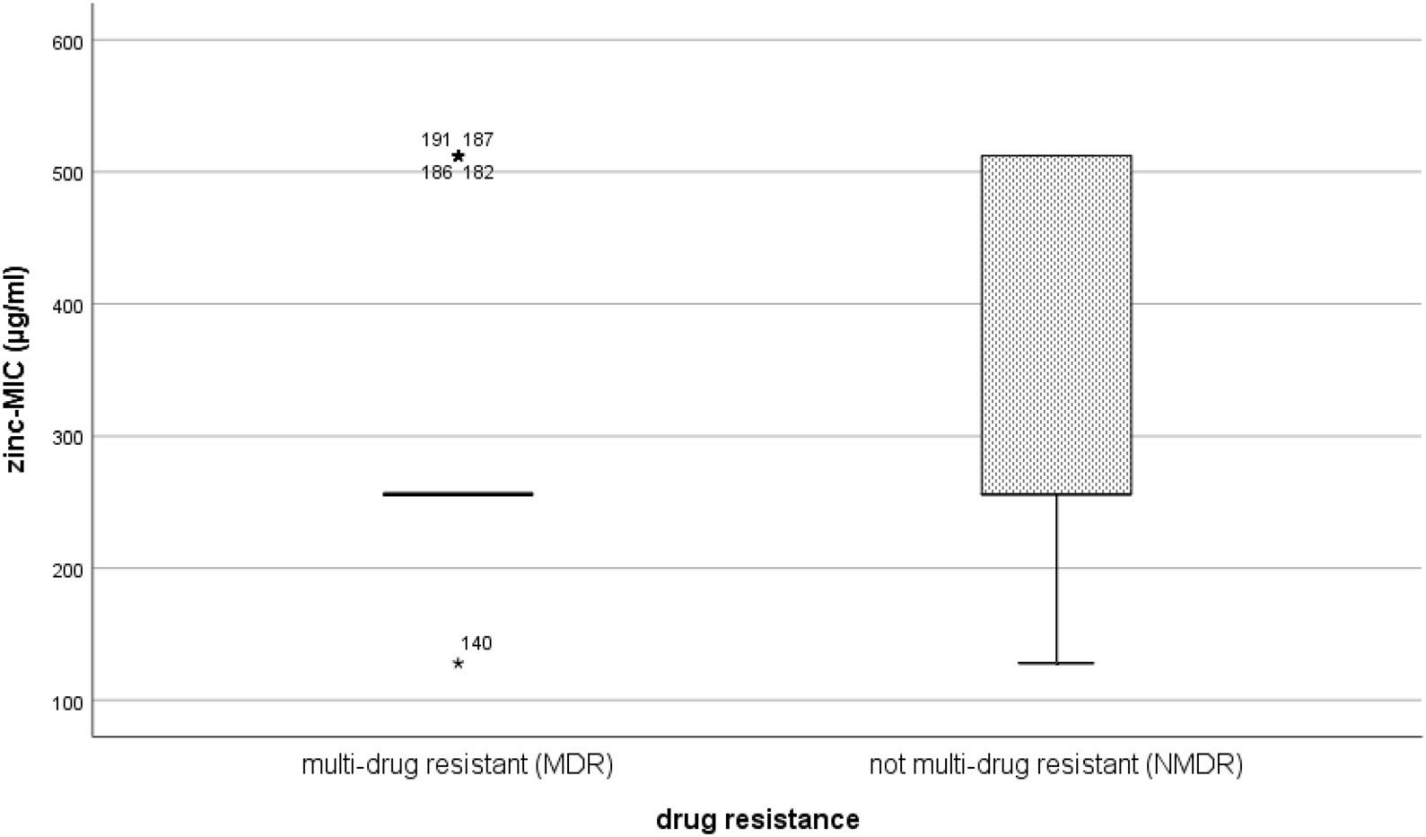
Comparison of MIC values for zinc; multi-drug resistant and not-multi-drug resistant isolates of both trials. A Mann–Whitney U test comparing 63 MDR isolates and 147 NMDR isolates (overall 210 isolates) showed no significant difference of zinc-MIC value between considered groups (P = 0.085)

The zinc tolerance data was not normally distributed (Kolmogorov–Smirnov test, P < 0.001). As shown in Fig. 2, there was no significant difference for the MIC of zinc between MDR and NMDR isolates (median_MDR_ = 256 µg/ml, median_not-MDR_ = 256 µg/ml P = 0.085).

**Fig. 3:**
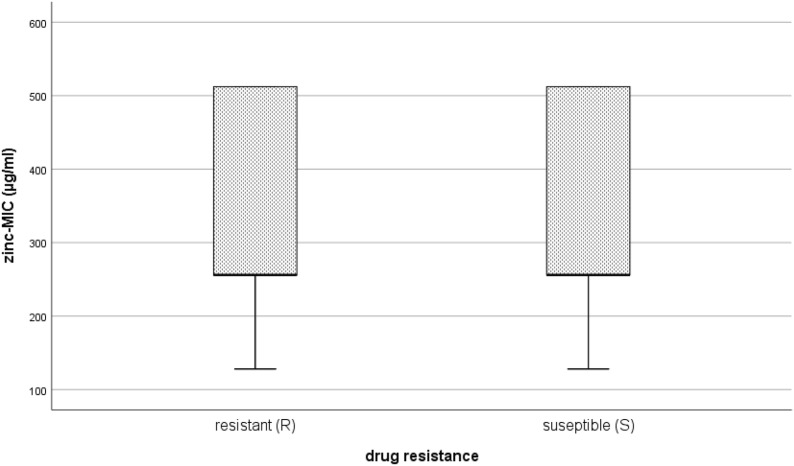
Comparison of MIC values for antimicrobial resistant (R) and -susceptible isolates (S). A Mann–Whitney U test comparing 124 resistant isolates and 86 susceptible isolates (overall 210 isolates) showed no significant difference of zinc-MIC value between considered groups (P = 0.107)

There was also no significant difference MIC values towards zinc of resistant isolates (R) compared to susceptible isolates (S) (median_resistant_ = 256 µg/ml, median_susceptible_ = 256 µg/ml, P = 0.107) (Fig. 3).

**Fig. 4:**
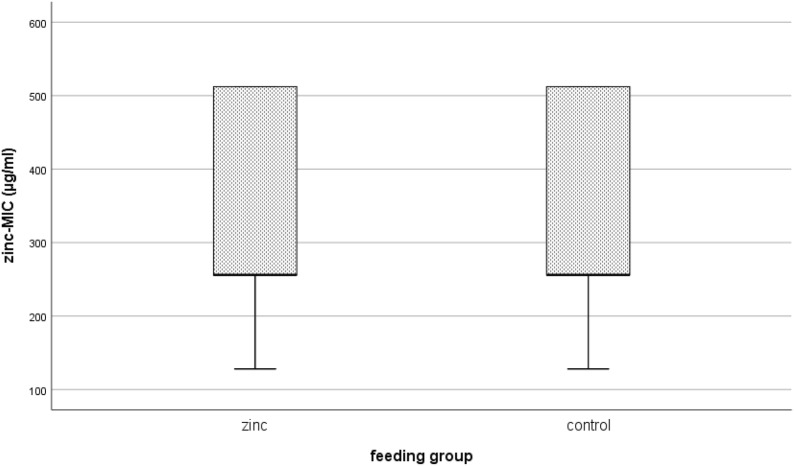
Comparison of MIC values for zinc; high-zinc supplementation group (zinc) and the background control (control) isolates from both trials. A Mann–Whitney U test comparing 114 isolates from zinc group and 96 isolates from control group (overall 210 isolates) showed no significant difference of zinc-MIC value between considered groups (P = 0.146)

Interestingly, as shown in Fig. 4, there was also no significant difference in the MIC values for zinc comparing isolates from the high-zinc supplementation group (median_zinc_ = 256 µg/ml) or control group (median_control_ = 256 µg/ml, P = 0.146).

### 3. Copper tolerance (MIC)

**Fig. 5:**
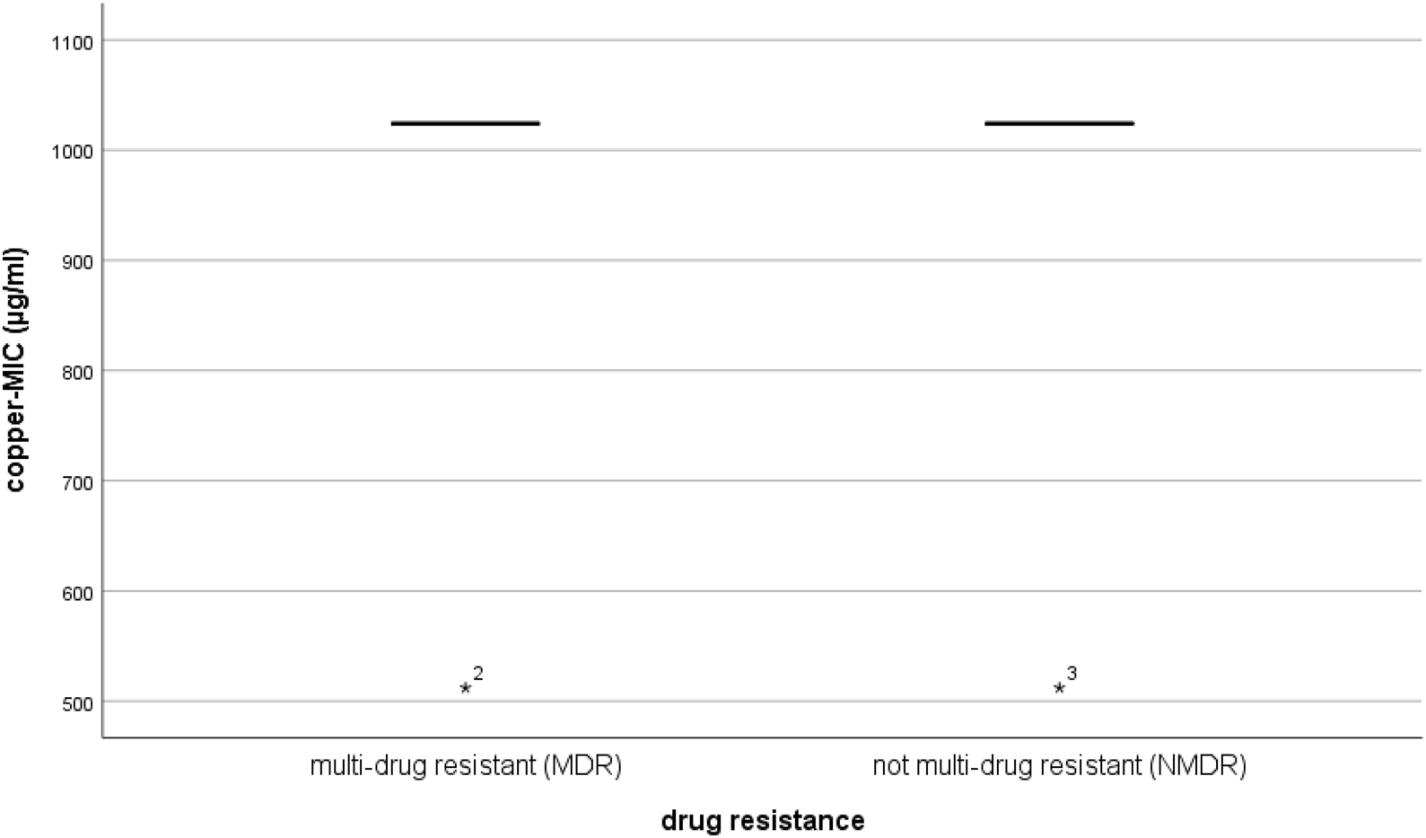
Comparison of MIC values for copper; multi-drug resistant and not multi-drug resistant isolates of both trials. A Mann–Whitney U test comparing 63 MDR isolates and 147 NMDR isolates (overall 210 isolates) showed no significant difference in the copper-MIC value between groups (P = 0.540)

All tested isolates in our experiment, with two exceptions, had MICs of 1024 µg/ml (~ 6.4 mM) for copper sulphate. No statistically significant difference in the MIC values towards copper comparing MDR (median_MDR_ = 1024 µg/ml) and NMDR (median_NMDR_ = 1024 µg/ml) isolates was observed (P = 0.540) (Fig. 5). There was also no significant difference in the MIC values for copper between resistant (R) and susceptible isolates, or isolates from the high-zinc supplementation group and control group (data not shown). There was no correlation between the zinc-MIC values and copper-MIC values (P = 0.593, correlation coefficient = − 0.037).

## Discussion

During two, independent animal trials, we observed an increase in multi-drug resistant (MDR) *E. coli* in isolates of piglets when fed with high concentrations of zinc. One possible explanation for this effect is a co-selection for heavy metal and antimicrobial resistance, as has been previously suggested [16, 19, 20, 25, 43]. To determine whether there is an association between MDR phenotype and phenotypic zinc tolerance, we screened both MDR and non-MDR (NMDR) isolates for the level of phenotypic zinc tolerance. Out of a total of 210 isolates selected from both zinc supplementation trials, 63 isolates (30%) were multi-drug resistant.

In this study, we determined two different classifications of antibiotic resistance. We compared multi-drug resistant (MDR) to non-multi-drug resistant isolates (NMDR) according to the definition of Schwarz et al*.* [44], as well as resistant isolates (R), defined as resistance to at least one antimicrobial agent, and susceptible (S) isolates, defined as not resistant to any antimicrobial agents. For both definitions of antimicrobial resistance, we obtained the same result. Isolates tested in this study are not the whole set of isolates derived from two previous studies. We also did not want to show differences in the number of multi-drug resistant strains. In contrary, we chose almost identical number of strains for this experiment to compare their zinc resistance and whether it correlates with their original MDR phenotype. Therefore, it should not necessarily be a significant difference between the number of MDR isolates from zinc and control-feeding groups as was determined in our previous studies.

When comparing susceptible isolates (S) to isolates harboring at least one (or more) resistances (R), we observed no significant difference (P = 0.107) in their zinc MIC values. In addition, the zinc MIC values for zinc of MDR *E. coli* and NMDR isolates also showed no significant difference, suggesting that there is no association between antimicrobial resistance and phenotypic zinc tolerance of these isolates.

The observed increase in MDR—*E. coli* during the zinc feeding trials is therefore not likely a result of co-selection of zinc and antimicrobial resistance. As proposed by Ciesinski et al. [18], the increase of multi-drug resistant isolates in swine treated with a high dietary zinc, is likely due to formation of a persistent population of resistant bacteria already present in the gut. Furthermore, we found no difference in zinc tolerance levels of isolates from zinc-treated groups in comparison to the control groups, suggesting that the overall zinc tolerance of *E. coli* in the gut of piglets therefore seems not be affected by zinc feeding.

There are no universal interpretative criteria for classification of *E*. *coli* resistance towards zinc, and studies determining the MIC values for heavy metal ions are scarce [45–48]. In this study, we used a custom-made plate for phenotypic zinc tolerance levels in *E*. *coli* and which include all inhibitory concentrations mentioned in previous studies in MIC assays [45].

Despite variations in the testing methods used in previous studies and our study, such as use of agar plates or broth micro-dilution, the medium used for growing bacteria, and the formulation of zinc used in the experiments, the biological upper cut-off of phenotypic zinc tolerance for the most of *E. coli* isolates in these studies were around 2–2.5 mM of zinc ion. This is in accordance with the reported MIC of 2.2 mM Zn 2+ for *E. coli* TG1 in LB-medium [47]. The highest reported concentration of zinc (Zn 2+) which could be tolerated by *E. coli* isolates in the literature was 5 mM [48].

To determine whether our findings were similar for other heavy metals, we also compared our isolates for copper tolerance. All tested isolates in our experiment, with two exceptions, had the same MIC values of 1024 µg/ml for copper sulphate (~ 6.4 mM). The highest MIC concentration of copper (Cu 2+) detected for *E. coli* isolates in prior studies was 10.5 mM. Our results indicated no difference between copper MIC values of MDR and NMDR isolates, suggesting that there is no association between phenotypic antimicrobial resistance and phenotypic copper tolerance of the isolates. Interestingly, we also observed no correlation between the zinc and copper MIC values of the same isolates.

Co-selection for antimicrobial and metal-resistance has been suggested in many studies [11, 39–41]. In many of these studies, co-resistance was not shown, but a co-existence of resistance was reported in the same bacteria. Nevertheless, it is believed that some metal and antimicrobial resistance genes are linked and co-resistance of antimicrobial and heavy metal resistant bacteria have been discussed in several studies as likely to arise through co-selection [22, 29–34].

These studies are mostly on genome level and several of studied antimicrobial and metal ion resistance genes are on plasmids [16, 32, 43, 49–51]. For example, the plasmids of *Salmonella abortus equi* were found to co-transfer antimicrobial resistance (ampicillin-resistance) and heavy metal resistance (As, Cr, Cd, Hg) genes in mating experiments with *E*. *coli* strains. *Salmonella* strains cured of the plasmids were found to be sensitive towards ampicillin and heavy metals [32]. In a genomic transcriptional study, Lee et al. [30] found up-regulation of the *mdtABC* operon after exposure to high levels of zinc which suggested a potential influence of metal stresses on bacterial resistance to antibiotics.

In a recent genomic study by Pal et al*.* [28], a total of 2522 fully sequenced bacterial genomes and 4582 plasmids were analyzed. The authors concluded from their large-scale study that plasmids have only a limited potential for horizontal transfer of biocides and metals resistance by co-selection.

Prior studies have also tested isolates at both the genomic and phenotypic levels. One such study showed co-regulation of resistance to heavy metals and carbapenems through the CzcR–CzcS system in *Pseudomonas aeruginosa* strain PT5. In that study, it was shown that a mutation in the CzcS sensor protein found in zinc and imipenem resistant isolates led to efflux pump CzcCBA overexpression and down-regulation of the OprD porin resulting in a co-selection for both increased zinc and carbapenem resistance [52]. In a series of retrospective studies screening *E. faecium* isolated from different species, it was found that *tcrB* (transfer copper resistance) and *ermB* (transfer macrolide resistance) genes were present on the same conjugative plasmid. However, the data did not demonstrate a co-selection between these two phenomena and the strong correlation between copper and macrolide resistance was found only in pig isolates. In addition, while the prevalence of macrolide resistance in isolates decreased during the years covered in the study, the prevalence of copper resistance among pig *E. faecium* isolates remained more or less the same. The authors argued that the reduction in the antimicrobial usage during this period lead to a decrease in antimicrobial resistance, whereas in the same time period the use of copper derivatives remained unchanged. Therefore, they concluded that copper exposure might not alone be sufficient to induce antimicrobial resistance and a strong selective pressure of macrolide administration should be present to select the antimicrobial resistance [40, 53, 54].

There are few experimental studies available evaluating the induction of antimicrobial resistance following metal exposure. Peltier et al*.* [55] investigated antimicrobial resistance to ciprofloxacin, oxytetracycline, and tylosin in zinc-activated sludge bioreactors. Zinc application alone did not affect zinc and antimicrobial resistance to ciprofloxacin and oxytetracycline. Increased antimicrobial resistance could be the result of co-exposure of zinc and antimicrobial agents. Berg et al*.* [56] found that strains isolated from soil treated with copper for 21 months were more resistant to both copper and indirectly antimicrobials compared to control plots.

In contrast to the above-mentioned studies in which co-selection was the subject of discussion, there are also studies reporting counter-selection of heavy metal and antimicrobial resistance [57]. Hölzel et al*.* [26] found that while exposure to zinc and copper increased the rate of β-lactam resistance in *E. coli*, the presence of mercury was associated with a lower rate of antimicrobial resistance.

## Conclusions

In summary, our results do not indicate a co-selection process of antimicrobial resistance and higher zinc tolerance in the MDR isolates of our feeding trials. An increase of *E. coli* more tolerant to zinc due to the feeding of high zinc concentrations as an explanation for the increase of multi-drug resistant isolates via co-selection can therefore be excluded. This seems to be also true for copper tolerance levels. These results would appear to argue against a co-selection mechanism for drug-resistance after zinc supplementation, since we did not find an association between antimicrobial resistance and phenotypic zinc/copper tolerance for the same isolates. We also found that zinc exposure did not have an effect on either zinc or copper phenotypic tolerance of the isolates.

An explanation for an increase in MDR isolates from piglets with high zinc dietary feeding in our previous studies could be that resistant bacteria to antimicrobial agents are more persistent to stresses such as zinc or copper exposure. Ciesinski et al*.* have argued that the increase in multi-drug resistant *E*. *coli* populations is associated with persistence of the resistant population under the influence of high dietary zinc, while in that study the total number of *E*. *coli* population had been decreased.

Another explanation might be that in the zinc-fed groups, zinc activates genes involved in metal ion resistance to deal with the metal ion load, and which might also be involved in antibiotic resistance, but this is a transient phenotypic zinc resistance. In accordance to this argumentation, Peltier et al*.* also found that zinc exposure increases resistance to antibiotics but had a minimal effect on zinc resistance [55]. In addition, the duration of experiments, co-exposure to both metal and antimicrobial agents and concentration of the substances could play role in either in vivo or in vitro-resistance studies.

Interpretation and analysis of resistance data based only on genetic data should be made carefully, a combination of both genetic and phenotypic resistance determinations is required, and it will also be important to show whether resistance could be developed in non-resistant isolates. The result of these types of studies could have implications for the prophylactic use of zinc in the field, i.e. pigs daily fed zinc to prevent infections.

## Methods

### Sample origin

**Fig. 6:**
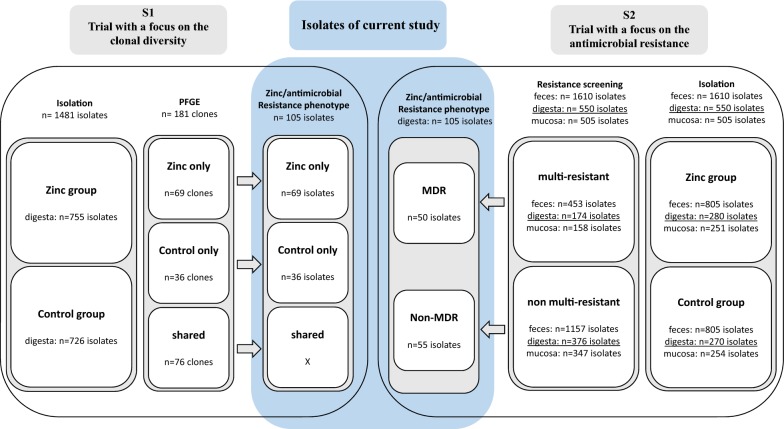
Schematic workflow of *E. coli* analyses. Number of isolates investigated in S1, S2 trials and the current study, design and focus of each study. *PFGE* pulsed-field electrophoresis, *MDR* multi-drug resistant, *non-MDR* non multi-drug resistant

A total of 210 *E. coli* isolates originally collected during two independent zinc feeding trials (S_1_ and S_2_) in 36 and 32 piglets respectively were used in this study (S_1_ = 105, S_2_ = 105) [18, 58]. All the experimental trials of these studies were approved by the local state office of occupational health and technical safety ‘Landesamt für Gesundheit und Soziales, Berlin’ (LaGeSo Reg. Nr. 0347/09 and LaGeSo Reg. Nr. 0296/13). The *E. coli* isolates were isolated from intestinal contents (digesta) on the 1st, 2nd and 4th weeks of both feeding trials. The first trial (S_1_) was a clonal study concentrating on the diversity of the *E. coli* analyzed via PFGE, which identified 105 clones from 1481 isolates in either only control or only zinc feeding groups independent of sampling time. In this study, one isolate from each of the 105 clones was tested. To obtain a comparable number of samples from the second feeding trial (S_2_), we randomly chose 105 isolates using representative random sampling method out of a total of 550 samples isolated from digesta [59]. The second feeding trial was performed with a selective culturing approach using CHROMagar Orientation plates supplemented with one of nine different antimicrobials as well as CHROMagar Orientation plates without supplementation to select resistant *E. coli* populations during the zinc treatment. Antibiotic concentrations in media plates were adapted from Guenther et al. [60] or are derived from the breakpoint concentrations of the Clinical and Laboratory Standards Institute [61, 62]. The schematic workflow of *E. coli* analyses (Fig. 6) shows the study design of previous and current studies.

In both trials, zinc oxide (Sigma Aldrich, Taufkirchen, Germany) was applied as a feed supplement to a high zinc feeding group (2000–2500 ppm) and background control (50–70 ppm). Further details of the animal trials can be found in the original publications [18, 58].

### Phenotypic antimicrobial resistance

All isolates were initially screened for their resistance profiles against ampicillin, chloramphenicol, gentamicin, streptomycin, tetracycline, cefotaxime, enrofloxacin, sulfamethoxazole/trimethoprim and imipenem (BD BBL Sensi-Disc Antimicrobial Susceptibility Test Discs, Becton-Dickinson, United States) according to the standards of the Clinical and Laboratory Standards Institute [63]. The results from the agar disc diffusion tests were confirmed using minimum inhibitory concentration (MIC) microdilution using cation adjusted Mueller Hinton II medium (Micronaut breakpoint plates, Genzyme Diagnostics, Rüsselsheim, Germany) according to CLSI standards (CLSI, 2008). Based on their resistance patterns these strains were stratified as multi-drug resistant or non-multi-drug resistant according to the definition of Schwarz et al. [44], as resistant (resistant to at least one antimicrobial agent) or susceptible (completely sensitive to the tested antimicrobials).

### Phenotypic zinc/copper resistance testing

Overnight cultures of all *E. coli* isolates were adjusted to McFarland Standard 0.5 (1.5 × 10^8^ CFU) and 50 µl of 1:200 dilution of adjusted suspensions in Mueller–Hinton broth (Roth, Karlsruhe, Germany) were used as inocula for incubations for 16 to 20 h at 35 °C in biocide and heavy metal microtiter-plates (Merlin, Bornheim-Hersel, Germany). The plates contained a wide range of concentrations of biocides/heavy metals in twofold dilution steps including 32 to 8192 μg/ml copper sulfate (COP) and 4 to 8192 μg/ml zinc chloride (ZKC) [45]. In our study, the minimal inhibitory concentration data of two heavy metals including copper sulfate and zinc chloride were collected. To prevent drying of the plates during incubation a sealing tape was used to seal the surface of the plate. After the incubation, the MIC for zinc was determined visually and reported as the growth breakpoint. *E. coli* ATCC25922 and ATCC10536 strains were used as reference strains for internal quality control.

### Statistical analysis

Statistical analysis was performed based on the combined datasets from both zinc feeding trials. Isolates were stratified irrespective of the zinc feeding either as multi-drug resistant (MDR) or non- multi-drug resistant (NMDR) isolates, as well as resistant (R) (at least one resistance) or susceptible (S) isolates. In addition, the isolates were subsequently grouped based on their origin from either high-zinc supplementation group (zinc) or the background control (control). Statistical analyses were performed using the SPSS software, version 25.0 (IBM, New York, NY, USA). The normal distribution of data was evaluated by a 1-sample Kolmogorov–Smirnov test. Mann–Whitney (non-parametric test) and chi-square tests were used for the analysis of data [64, 65]. The correlation between zinc tolerance and copper tolerance was calculated using Spearman rank correlation test (non-parametric correlation) [66]. The non-normally distributed data are shown as the median ± standard deviation (SD), and P < 0.05 was considered statistically significant.

## Data Availability

The datasets used and/or analyzed during the current study are available from the corresponding author on reasonable request.
